# Do systematic reviews address community healthcare professionals’ wound care uncertainties? Results from evidence mapping in wound care

**DOI:** 10.1371/journal.pone.0190045

**Published:** 2018-01-10

**Authors:** Janice Christie, Trish A. Gray, Jo C. Dumville, Nicky A. Cullum

**Affiliations:** 1 Division of Nursing, Midwifery and Social Work, School of Health Sciences, Faculty of Biology, Medicine and Health, University of Manchester, Manchester Academic Health Science Centre, Manchester, United Kingdom; 2 Research and Innovation Division, Central Manchester University Hospitals NHS Foundation Trust, Manchester Academic Health Science Centre, Manchester, United Kingdom; TNO, NETHERLANDS

## Abstract

**Background:**

Complex wounds such as leg and foot ulcers are common, resource intensive and have negative impacts on patients’ wellbeing. Evidence-based decision-making, substantiated by high quality evidence such as from systematic reviews, is widely advocated for improving patient care and healthcare efficiency. Consequently, we set out to classify and map the extent to which up-to-date systematic reviews containing robust evidence exist for wound care uncertainties prioritised by community-based healthcare professionals.

**Methods:**

We asked healthcare professionals to prioritise uncertainties based on complex wound care decisions, and then classified 28 uncertainties according to the type and level of decision. For each uncertainty, we searched for relevant systematic reviews. Two independent reviewers screened abstracts and full texts of reviews against the following criteria: meeting an a priori definition of a systematic review, sufficiently addressing the uncertainty, published during or after 2012, and identifying high quality research evidence.

**Results:**

The most common uncertainty type was ‘interventions’ 24/28 (85%); the majority concerned wound level decisions 15/28 (53%) however, service delivery level decisions (10/28) were given highest priority. Overall, we found 162 potentially relevant reviews of which 57 (35%) were not systematic reviews. Of 106 systematic reviews, only 28 were relevant to an uncertainty and 18 of these were published within the preceding five years; none identified high quality research evidence.

**Conclusions:**

Despite the growing volume of published primary research, healthcare professionals delivering wound care have important clinical uncertainties which are not addressed by up-to-date systematic reviews containing high certainty evidence. These are high priority topics requiring new research and systematic reviews which are regularly updated. To reduce clinical and research waste, we recommend systematic reviewers and researchers make greater efforts to ensure that research addresses important clinical uncertainties and is of sufficient rigour to inform practice.

## Introduction

The commonest types of complex wounds are venous leg ulcers, complex surgical wounds, pressure ulcers and foot ulcers (due to diabetes and other causes) [1,2]. A comprehensive survey in one large UK city estimated that 1.47 people per 1000 (95% CI 1.38 to 1.56 per 1000) have a complex wound at any time [1]. The care of people with complex wounds is costly because wound healing is protracted [3], with for example, long term wound management accounting for an estimated 5.5% of all UK National Health Service (NHS) expenditure (estimate based on records of primary and secondary care consultations within family doctor records in Wales) [4]. Delayed healing also has a negative impact on people’s quality of life [1,5]. As the world’s population ages [6] health resources needed for managing complex wounds and the implications for society will continue to rise; therefore, clinically- and cost-effective wound management is a pressing international health concern [7].

The quality of evidence derived from wound care trials tends to limited due to drawing on: underpowered studies that have small numbers of participants and/or few event numbers, short-term follow-up and sub-optimal use of research methods and outcomes [8]. Robust research in wound care is possible and has been undertaken but obtaining competitive funding can be challenging. In common with most areas of healthcare, therefore, there is much clinical uncertainty in wound care [1,9]; this means decision makers are often unclear regarding the best courses of action to achieve a desirable clinical or service outcome [10]. Clinical uncertainties can contribute to the use of ineffective treatments and patient harm [11] with variations in practice leading to inequalities [12] and wasted healthcare resources [13]. Various factors contribute to uncertainties in clinical practice, such as lack of scientific data, organisational issues (changes in service provision or procedures, poor leadership and financial constraints) and patient uncertainty (caused by the uniqueness of each patient’s situation and/or professional-patient interactions and relationships) [14]. Uncertainty can affect any type of healthcare decision that practitioners may make, such as assessment, diagnosis, intervention, communication, referral, service delivery and organisation, and information-seeking [15]. It has been suggested that increasing the availability of high quality research evidence on topics relevant to patients and healthcare professionals is one way of tackling uncertainties that arise from lack of scientific data [16]; such progress would this requires that the right research is commissioned and conducted.

It has been estimated that about 80% of biomedical research is wasted [17]; wastage can occur at any point from research funding through to research dissemination. To avoid such waste, the commissioning and conduct of healthcare research needs to be better aligned with the uncertainties that research users deem to be important [18]. It is also essential that any research conducted is as high quality as possible to ensure that findings are robust; thereby reducing levels of clinical uncertainty. Ideally research questions should be identified and prioritised through a collaborative process with relevant stakeholders [19]. This type of work is being increasingly undertaken to establish and prioritise key healthcare uncertainties [20–22].

Uncertainty identification and priority setting has been undertaken for pressure ulcers, general wound or burns care [1,23,24]; involving patients, carers and healthcare professionals [1] or healthcare professionals alone [23,24]. More recently, as part of the National Institute for Health Research Collaborations for Leadership in Applied Health Research and Care (NIHR-CLARHC) in Greater Manchester, we asked healthcare professionals, namely: community nurses, podiatrists, specialist nurses and clinical managers to generate, prioritise and rank clinical uncertainties related to wound care [25]. The exercise generated 28 prioritised wound care uncertainties; these were real ‘working day’ clinical decision uncertainties as identified and prioritised by the healthcare professionals using a consensus-based nominal group technique.

After harvesting decision uncertainties it is also necessary to establish whether elicited uncertainties are ‘genuine’ uncertainties (i.e., not already answered by research) or areas where relevant research findings exist but are not known. This paper describes the process by which we mapped existing evidence to the 28 wound care uncertainties gathered from healthcare professionals. We followed a pragmatic and generic evidence-based practice approach [26,27] in which we searched for and then examined the quality of the evidence. We sought only research evidence in the form of systematic reviews since they provide comprehensive, pre-appraised and condensed relevant research evidence for healthcare professionals [28,29]. We took the view that where we found relevant, contemporary systematic reviews drawing on robust evidence that reduced or eradicated the expressed uncertainty; this might indicate a dissemination or implementation priority. Conversely, if we found: no, irrelevant, out-of-date systematic reviews or systematic reviews containing weak or no primary research evidence, then this would suggest we had uncovered a priority for evidence synthesis or primary research.

## Methods

We mapped the nature of existing systematic review evidence for the 28 highest priority wound care uncertainties expressed by community-based healthcare professionals in the UK. We conducted this in two stages; firstly, we broadly classified our priorities into types and the level of clinical decision. We then systematically searched the literature for reviews using pre-specified criteria to identify up-to-date systematic reviews containing high quality evidence addressing any of the 28 uncertainties.

### Classification of wound care uncertainties

We classified the wound care uncertainties using an adaptation of the decision typology of McCaughan et al [15], which classifies clinical decisions as concerning: assessment, diagnosis, intervention, communication, referral, service delivery and organisation and information-seeking. Our adaptation of the typology involved separating it into two components: the type of decision (assessment, diagnosis, intervention, communication, referral, or information-seeking); and the level of decision (wound, patient or service), see Table 1 for definitions. These levels of decision-making were adapted from social-ecological theory [30,31].

**Table 1:** Decision typology definitions.

Category	Definition
***Type of decision*:**	
Assessment	Deciding how to determine if signs and/or symptoms are present; deciding which signs and/or symptoms to search for
Diagnosis	Deciding what diagnostic label is indicated by presenting signs and symptoms
Intervention	Deciding what intervention to offer/use, and/or when
Communication	Deciding how to give or gain information
Referral	Deciding who to refer to and/or when
Information-seeking	Deciding if pursuing/not pursuing further information before making a decision
***Level of decision*:**	
Wound	Making decisions about wound care
Patient	Making decisions about patient care
Service	Making decisions about service organisation, delivery and management

### Searching for and evaluating evidence

**Fig 1:**
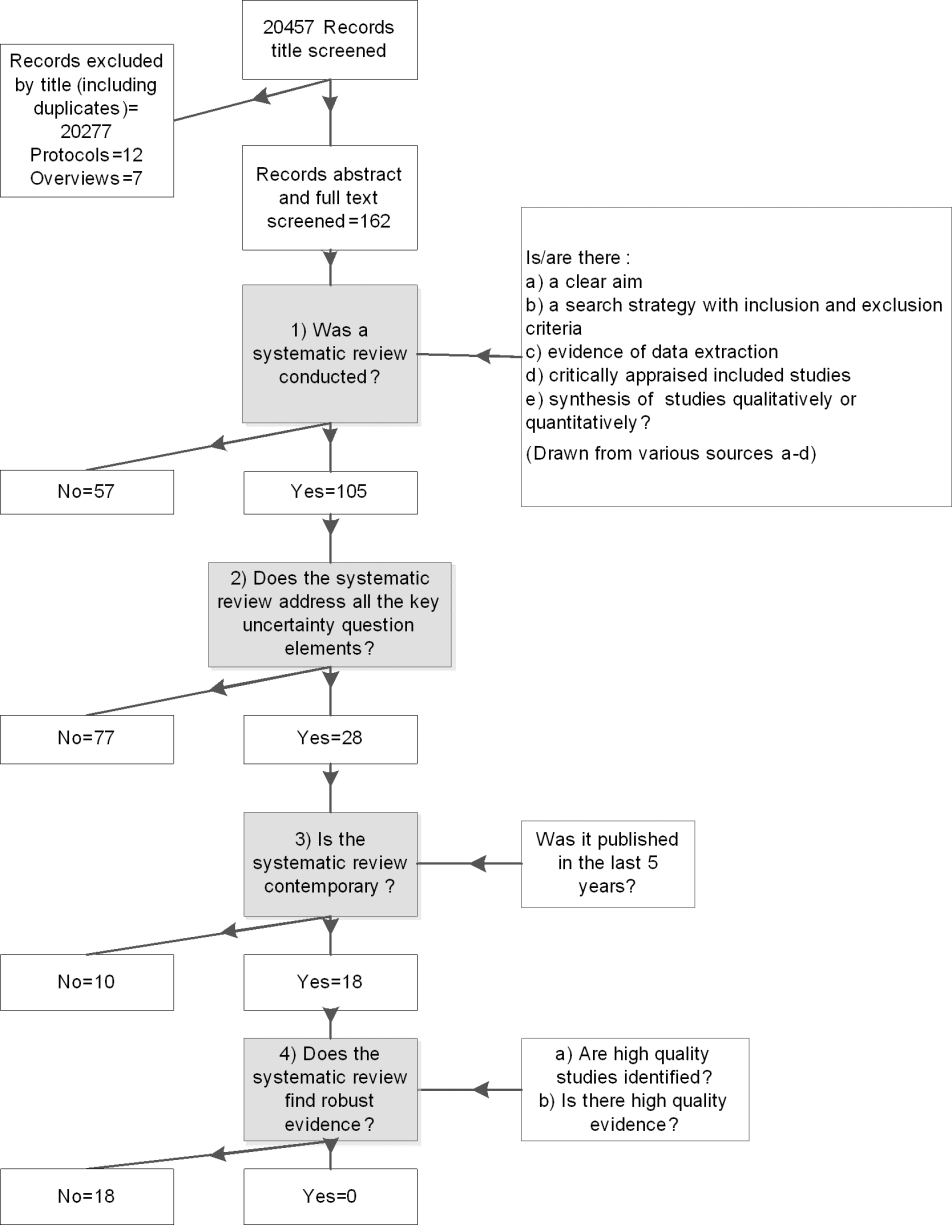
Evaluation process and criteria. We present the total number of review records screened, the four criteria used for screening abstracts/full papers, and the number of reviews meeting/not meeting each criterion. a,NHS centre for reviews and dissemination (2002) The Database of abstracts of reviews of effects (DARE). Effectiveness Matters: 6:1–4. b,Hemingway P, Brereton N (2009) What is a systematic review?. What is? series: Hayward Medical Communcations. c,Moher D, Liberati A, Tetzlaff J, Altman DG, The PG (2009) Preferred Reporting Items for Systematic Reviews and Meta-Analyses: The PRISMA Statement. PLoS Med 6: e1000097. d,Shea BJ, Grimshaw JM, Wells GA, Boers M, Andersson N, Hamel C, et al. (2007) Development of AMSTAR: a measurement tool to assess the methodological quality of systematic reviews. BMC Med Res Methodol 7: 10.

For each of the 28 uncertainties we systematically searched for reviews and assessed them against four criteria in sequence, namely: whether they were clearly defined systematic reviews according to pre-specified conditions; and when so, if they were sufficiently relevant to the uncertainty; then whether they were sufficiently up-to-date (published or updated in the last 5 years) and if the systematic review identified high quality research evidence using a clear and appropriate criteria or framework (for example GRADE [32]). Detailed specifications of each of the four criteria are presented in Fig 1.

We did not place language or year of publication limitations on our searches (as we wanted to determine if wound care systematic reviews that met our uncertainties existed but had not been updated). Overviews of systematic reviews were not eligible; instead we searched the reference lists of overviews for relevant systematic reviews that may not have been identified in our searches. We also noted any systematic review protocols returned in our searches.

We searched the Cochrane Database of Systematic Reviews (CDSR) up to 8th August 2016 and the Database of Abstracts of Reviews of Effects (DARE) to 31^st^ March 2015 via the Cochrane Library; and PubMed using the ‘systematic[sb]’ search filter until 8^th^ August 2016. We developed 26 bespoke searches for each of the 28 uncertainties (S1 Table, using two search strategies twice for uncertainties with shared key concepts); we developed our searches using a PICO format [33] where possible. If an uncertainty was broad and ill-defined we focused on the most relevant or common scenario associated with the question.

One reviewer ran the searches and then screened the results by title to remove any obviously irrelevant hits. The abstracts of potentially relevant reviews were screened by two people independently. Those reviews thought to be relevant based on title and abstract were obtained as full-text and a final decision on inclusion was based on this. Any disagreement regarding eligibility was resolved by reference to a third reviewer. We recorded search results, screening and evaluation processes in an Excel (Microsoft Office 2010) spread sheet.

## Results

The 28 uncertainties, classified by type and level of decision, are listed in order of highest ranking with their priority ranking score given by the healthcare professionals (see Table 2). We identified only assessment, diagnostic and intervention uncertainty decision types and these were targeted at all three levels of decision-making (wound, patient and service delivery). The majority (24/28; 86%) of the healthcare professionals’ uncertainties concerned intervention decisions, though the two assessment decisions were prioritised highly. Wound level uncertainties were the most frequent (15/28). Overall, there were ten service level decision uncertainties; these tended to be prioritised highly (eight of the 13 highest ranked uncertainties (numbered 1–11) concerned service level decisions).

**Table 2:** Classification of healthcare professionals’ uncertainties.

Number	Uncertainty	Ranking	Type of decision	Level of decision
**1**	Does patient involvement in their dressing changes improve outcomes or increase negative outcomes?	1	Intervention	Patient
**2**	What is the most reliable and valid method of grading pressure ulcers?	1	Assessment	Wound
**3**	Would standardising wound assessments and tools across NHS settings improve staff productivity and patient outcomes?	3	Assessment	Service
**4a**	How does nursing and/or professional skill mix influence wound outcomes in community settings?	3	Intervention	Service
**4b**	What training is required to best manage patients with complex wounds?		Intervention	Service
**5**	Do integrated team-based interventions aimed at better communication and collaborative working, improve patient outcomes?	3	Intervention	Service
**6**	Does continuing professional development in wound care improve the quality of care and patient outcomes compared with no annual update?	3	Intervention	Service
**7**	What effects do electronic patient records have on patient and service outcomes across a wound care service compared to paper records?	3	Intervention	Wound
**8**	Which treatments are most effective for over granulation?	3	Intervention	Service
**9**	What is the most clinical and cost-effective criteria for referring to specialist services (e.g. tissue viability/podiatry) to ensure appropriate use of resources and referral time?	3	Intervention	Service
**10a**	How do we differentiate between diabetic foot wounds and pressure ulcers?	3	Diagnosis	Wound
**10b**	Does this influence management and outcomes?		Intervention	Wound
**11**	Do patients with venous leg ulceration heal quicker when treated in a dedicated leg ulcer clinic compared with general community clinics?	3	Intervention	Service
**12**	What are the effects of different cleansing agents on infection and healing of wounds in community settings?	12	Intervention	Wound
**13**	What are the clinical and cost effective methods for managing an excess of wound exudate?	12	Intervention	Wound
**14**	Does sharp debridement speed up wound healing in chronic wounds compared with dressings (HCL, hydrogels etc)?	12	Intervention	Wound
**15a**	How should we identify where biofilm is impeding wound healing?	12	Diagnosis	Wound
**15b**	What is the best way to manage a biofilm?		Intervention	Wound
**16**	How do we promote adherence to interventions and health behaviours in people at high risk of foot problems?	12	Intervention	Patient
**17**	Do anti-microbial containing wound dressings heal infected wounds more quickly than oral antimicrobials?	12	Intervention	Wound
**18**	Does a prescribed two week treatment plan, using the same type of dressing, affect healing outcomes versus ad-hoc dressing selection?	12	Intervention	Wound
**19**	What is the best way of cleaning venous leg ulcers in terms of promoting healing and preventing infection?	12	Intervention	Wound
**20**	Do psychological interventions (i.e., aimed at changing health beliefs and behaviours) improve the healing/reduce the incidence of ulcers on the feet of people with diabetes?	20	Intervention	Patient
**21**	How can accurate detection of clinical infection be facilitated across different skill mixes?	20	Intervention	Service
**22**	What should be used for infected wounds when the bacteria are resistant to antibiotics?	20	Intervention	Wound
**23**	Does off-loading for people with foot wounds Improve wound healing compared with usual (or increased) activity?	23	Intervention	Wound
**24**	What impact do walk in centres have on patients outcomes versus treatment room clinics?	23	Intervention	Service
**25**	Does stopping packing a sinus wound when it has healed to 1cm depth and then treating with medical honey speed wound healing compared with usual care?	23	Intervention	Wound

Uncertainty questions with identification number are presented with priority ranking given by healthcare professionals; they are also classified by type and level of decision. Uncertainties 4, 10 and 15 consisted of two questions which are considered separately in this paper; i.e. a total of 28 uncertainties are presented.

We screened 20,457 record titles from 26 searches for the 28 uncertainties (Fig 1 and Table 3).

**Table 3:** Search results.

Uncertainty	Total number of records screened	Records removed following title screen, de-duplication and removal of protocols/ overviews	Reviews screened by abstract and full text
1	22	22	0
2	183	178	5
3	1430	1418	12
4a	398	395	3
4b	1684	1684	0
5	3353	3336	17
6	532	528	3
7	2563	2546	17
8	129	129	0
9	174	169	5
10a	70	65	2
10b			3
11	47	43	4
12	248	239	9
13	63	61	2
14	696	683	13
15a	42	37	3
15b			3
16	306	295	11
17	770	760	10
18	3633	3629	4
19	16	14	2
20	130	128	2
21	3161	3152	9
22	178	175	3
23	331	321	10
24	137	132	5
25	162	157	5

Number of review records identified through the Cochrane Library and PubMed per uncertainty, records removed following title screen and screened by abstract and full text.

We identified a total of 162 potential systematic reviews for full text screening regarding 28 healthcare professionals’ uncertainties (see Table 4). In total, 57 of 162 reviews were not systematic; of the remaining 106 systematic reviews only 28 sufficiently addressed the uncertainty. Of the 28 relevant, systematic reviews, only 18 were up-to-date (published since 2012) and none identified high quality research evidence.

**Table 4:** Number of reviews screened and retained following application of each criterion.

Uncertainty	Decision type	Decision level	Reviews screened by abstract/full text	1.Systematic review	2.Addresses uncertainty	3.Published in last 5 years	4.Identifies robust evidence
1	Intervention	Patient	0	0	0	0	0
2	Assessment	Wound	5	2	1	1	0
3	Assessment	Service	12	10	0	0	0
4a	Intervention	Service	3	3	0	0	0
4b	Intervention	Service	0	0	0	0	0
5	Intervention	Service	17	11	4	2	0
6	Intervention	Service	3	2	0	0	0
7	Intervention	Service	17	6	1	0	0
8	Intervention	Wound	0	0	0	0	0
9	Intervention	Service	5	4	0	0	0
10a	Diagnosis	Wound	2	0	0	0	0
10b	Intervention	Wound	3	1	0	0	0
11	Intervention	Service	4	4	0	0	0
12	Intervention	Wound	9	4	4	2	0
13	Intervention	Wound	2	2	0	0	0
14	Intervention	Wound	13	11	1	1	0
15a	Diagnosis	Wound	3	0	0	0	0
15b	Intervention	Wound	3	0	0	0	0
16	Intervention	Patient	11	11	7	4	0
17	Intervention	Wound	10	7	1	0	0
18	Intervention	Wound	4	4	0	0	0
19	Intervention	Wound	2	2	2	2	0
20	Intervention	Patient	2	1	0	0	0
21	Intervention	Service	9	6	0	0	0
22	Intervention	Wound	3	3	1	1	0
23	Intervention	Wound	10	8	5	4	0
24	Intervention	Service	5	3	0	0	0
25	Intervention	Wound	5	1	1	1	0
Total			162	106	28	18	0

## Discussion

### Key findings

We have mapped the availability of relevant, up-to-date systematic reviews against 28 wound care decision uncertainties identified and prioritised by community-based healthcare professionals (nurses, podiatrists and managers). In the final stage of our mapping process we assessed the quality of primary research evidence identified in each systematic review and judged the extent to which it closed the expressed uncertainty (i.e. “answered the question”). A previous initiative, the Global Evidence Mapping (GEM) Initiative, considered evidence for Traumatic Brain and Spinal Cord Injuries [34] using a three step approach: 1) developing searchable questions (through engaging stakeholders and a broad literature review) 2) searching for and selecting relevant studies 3) extracting data about interventions and the studies; our uncertainties were not influenced by searching for existing research in advance. Thus, this is the first wounds research priority setting exercise that explicitly maps the available evidence against practitioner priorities in this way, identifying where systematic reviews and new primary research is needed.

After extensive searching we found that none of the 28 wound care uncertainties are resolved by good quality primary research evidence found within up-to-date systematic reviews. Following thorough scrutiny we found there were 19 important uncertainties with no relevant, up to date systematic reviews. While we identified 18 up-to-date, systematic reviews relevant to 9/28 uncertainties, none of these reviews identified high quality research evidence that answered the question. We did not, therefore, find that healthcare professionals were unaware of good wound care evidence (i.e. we did not identify wound care research knowledge transfer or implementation gaps). Instead, we identified a lack of systematic reviews and high quality primary research evidence; while not explicitly linked with evidence to meet clinical uncertainties, a lack of good quality wound care research has been previously reported [8]. Thus, we identified 28 areas which in addition to being clinical uncertainties and priorities are also wound care research and systematic review priorities requiring new primary research.

### Developing the current systematic review evidence base

Our search and screening originally identified 162 review articles of which 57 were rejected as not meeting our pre-determined definition of a ‘systematic review’. Most commonly these rejected reviews either did not have an explicit, comprehensive search strategy with evidence of systematic data extraction or evidence of critical appraisal of included studies. Some of these ineligible reviews were entitled ‘systematic reviews,’ highlighting that readers should be aware of misleading labelling of wound care publications and need to be able to distinguish systematic from other forms of review. It should also be noted that both “unsystematic” and out of date systematic reviews have the potential for producing misleading information for busy practitioners who do not have the resources to undertake full critical appraisals of the information they need to inform decision-making. The issue of review mislabelling also has significance for journal editors who should ensure that reviews they accept for publication and label as ‘systematic reviews’ adhere to PRISMA reporting guidelines [35] and meet minimum methodological criteria such as those we used.

We were unable to find any systematic review for six uncertainties (*the impact of patient involvement in wound care; training required to best manage patients with complex wounds; differentiation of diabetic foot and venous leg ulcers; treatments for wound over-granulation; diagnosis of biofilm* and *treatment of wound biofilm)*. These areas represent ‘desert’ priorities where the reason why there are no systematic reviews is unknown, but potentially there may be no primary research in the area to act as a catalyst for review production. In such circumstances even an empty systematic review can be helpful (when not misinterpreted as evidence of no effect) as empty reviews can clearly articulate to healthcare professionals that no research evidence exists, and to research funders and commissioners that research is needed [36].

Once a review has been undertaken, it needs to be updated as new research becomes available (such an approach is advocated by Cochrane [37]). We identified that 36% (10/28) of the relevant, systematic reviews we found were published more than five years ago.

### The need for high quality primary research

We classified the 28 complex wound care uncertainties identified by healthcare professionals in accordance with the type and level of decision. Through this process we found that most uncertainties concerned decisions about interventions (24 of 28); this concurs with previous evidence gathered from nurses in hospital and community settings which concluded that most of their clinical judgements concerned selection of interventions [15,38]. Given that most of our uncertainties related to interventions this indicates that more good quality randomised controlled trials, in particular, are required. Research commissioners and researchers need to ensure that robust wound care research is designed, funded and undertaken to avoid research and clinical waste.

We already know that there are deficiencies in the randomised controlled trials conducted about wound treatments [8]. Even for the small number (11%, 18/162) of good, up-to-date and relevant systematic reviews we found, none identified high quality research evidence.

We also uniquely identified that while most uncertainties concerned wound level decisions, service level decisions tended to have the highest priority: the reasons for this are likely to be multi-factorial. One partial explanation may be the importance of service delivery and management decisions in wound care [39,40]. Healthcare professionals may also feel service-level uncertainties more acutely than uncertainties relating to treatments, where personal and peer experiential knowledge can be used to mitigate a lack of research evidence.

While we identified 75 reviews for ten service level uncertainties, only five were relevant and systematic. Cochrane Effective Practice and Organisation of Care [41] has developed expertise in conducting service level systematic reviews [42], and greater use of these methodologies could help reduce the deficit in good quality wound care service-level reviews. Thus, we suggest that more service level intervention research and better quality service level systematic reviews need to be commissioned to support improvements in healthcare service access, provision and outcomes.

### Co-production of research evidence

Nearly 73% (77/106) of the systematic reviews we found did not sufficiently match the key elements of the healthcare professionals’ uncertainties and this raises essential questions about the nature of research evidence and who is involved in producing it, as ensuring the relevance and applicability for clinical practice of research is essential in order to maximise its value and avoid research waste [42]. Researchers and practitioners can be viewed as distinct communities of practice, each with their own, distinct, forms of tacit knowledge [43] and it is known that there can be a mismatch between how healthcare professionals view a clinical issue and how it is interpreted by researchers [19].

Indeed it has been suggested that the very process of identifying and prioritising researchable questions is part of the researchers’ tacit knowledge [43]. In order to ensure that both primary research and systematic reviews properly reflect important aspects of clinical uncertainty we need to either develop more clinical academics, who are fully engaged in both practice and research *or* develop meaningful collaborations *between* practitioners and researchers in order to co-produce research evidence (or a combination of the two) such as discussed by Heaton [44]. It is this latter model of co-production that we are pursuing within the wounds programme of the Greater Manchester CLAHRC. We also note the importance of eliciting uncertainties from service users and informal carers which we plan to do separately. Cochrane Wounds, a major producer of systematic reviews in wound care [45], has a policy of involving healthcare professionals as authors in their reviews to ensure clinical relevance. The uncertainties described here have directly influenced Cochrane Wound’s prioritisation of review topics.

When considering and prioritising clinical uncertainties it is important to consider that some staff may feel relatively ‘certain’ about some clinical decisions where research evidence is uncertain. This, in turn, might impact on how uncertainties are prioritised. For example, treatment clinical decisions might be informed by local policies and tradition rather than research knowledge [13] or advertising and other company information which is often not well supported by evidence [46]. In consequence, healthcare professionals may assume (based on policy, tradition or advertising) that the research evidence is less uncertain than it actually is. We suggest, therefore, that it is important to triangulate robust systematic review findings with clinical data to explore potential unexpressed and unrecognised clinical uncertainties.

### Limitations

We acknowledge some limitations regarding this paper. The clinical wound care uncertainties were generated by healthcare managers and practitioners in one locality of the UK and did not include the voices of patients and service planners. Nonetheless, given that we were unable to identify robust, research-based information on the priority areas in the international literature; and given their nature, we think it is likely these questions have relevance nationally and probably internationally (although the prioritisation may vary). The uncertainties of service planners and patients are also important and investigation of these stakeholders’ uncertainties needs further investigation. We propose that our mapping method, as presented in this paper, is an applicable adjuvant to such work.

Our mapping used simple search strategies which may have missed some evidence accessible through other unsearched databases; however, we searched databases through which we were most likely to identify international and quality healthcare systematic reviews. In determining if something addressed the uncertainty question directly or indirectly a measure of judgement required in deciding how much of the uncertainty was answered; however, we increased the reliability of our judgements by a consensus approach and dialogue with fellow investigators.

We acknowledge that we have only included systematic reviews and that some clinical guidelines are based on systematic review evidence undertaken during the guideline development process. We attempted to review systematic reviews that were part of guidelines and identified 44,000 guidelines relevant to the 28 uncertainties and then drew a purposeful sample of the first three, highest prioritised uncertainties (1 wound level intervention, 1 patient level assessment and 1 service level assessment). We found that none of the 100 guidelines we evaluated from 13000 search results for these 3 uncertainties had identified relevant, contemporary, high quality systematic review evidence; therefore, we conclude our findings are robust.

Our uncertainties were only collected from one locality within the UK; however, we contend that the uncertainties identified can apply to other complex wound care settings and are not addressed by internationally accessible systematic reviews. Nonetheless, the ranking of wound care uncertainties may vary between localities and should be verified in other settings. Finally we note that some uncertainties may have been addressed by primary research which are yet to be included in an up-to-date systematic review.

## Conclusion

The currently available systematic reviews do not address community healthcare professionals’ wound care uncertainties. Whilst good quality systematic reviews have been conducted, many uncertainties remain and further rigorous reviews are required to meet demand. This is the first study that mapped and evaluated complex wound care uncertainties identified by healthcare professionals; and the first to compare healthcare professionals’ prioritised wound care uncertainties against existing systematic review evidence, through development of a decision-making typology and evidence mapping. This paper generates insight for researchers and commissioners of wound care research to inform the development and commissioning of meaningful research that avoids research waste. The methods presented here can also be used to assess other types of the research evidence or evidence about uncertainties generated in other healthcare fields. Our evidence mapping also generates useful knowledge for educators and healthcare managers about the types of wound care decisions that currently do not have a strong, synthesised and appraised research-base; where healthcare professionals and patients may need guidance until a better wound care evidence-base is established.

## Supporting information

S1 Table Uncertainty search strategies for Cochrane Library and PubMed. (DOCX)
