# Supplementary material for: Do systematic reviews address community healthcare professionals’ wound care uncertainties? Results from evidence mapping in wound care
Source: PLoS One. 2018 Jan 10;13(1):e0190045. doi: 10.1371/journal.pone.0190045 (PMC5761849; doi:10.1371/journal.pone.0190045)
Supplement: S1 Table — (DOCX) [file pone.0190045.s001.docx]

|  | **Uncertainty** | **Cochrane Library search strategy** | **PubMed search strategy** |
| --- | --- | --- | --- |
| 1 | Does patient involvement in their dressing changes improve outcomes or increase negative outcomes? | ((patient* NEXT involv*) OR (self NEXT manag*) OR (self-manag*) OR (self NEXT car*) OR (self-car*) OR (self NEXT monitor*) OR (self-monitor*)) AND dressing* | systematic[sb] AND (patient involv* OR self manag* OR self-manag* OR self care OR self-care OR self-caring OR self caring OR self monitor* OR self-monitor*) AND dressing* |
| 2 | What is the most reliable and valid method of grading pressure ulcers? | ((bed NEXT sore*) OR (pressure NEXT sore*) OR (pressure NEXT ulcer*) OR (decubitus NEXT ulcer*)) AND (grad* OR classif* OR stag*) | systematic[sb] AND (bed sore* or pressure sore* or pressure ulcer* or decubitus ulcer*) AND (grading OR grade* OR stag* OR classif*) |
| 3 | Would standardising wound assessments and tools across NHS settings improve staff productivity and patient outcomes? | ((skin NEXT ulcer*) OR (foot NEXT ulcer*) OR (diabetic NEXT foot) OR (diabetic NEXT feet) OR (leg NEXT ulcer*) OR (varicose NEXT ulcer*) OR (venous NEXT ulcer*) OR (stasis NEXT ulcer*) OR (arterial NEXT ulcer*) OR ((ischaemic or ischemic) NEXT (wound* or ulcer*)) OR (bed NEXT sore*) OR (pressure NEXT sore*) OR (pressure NEXT ulcer*) OR (decubitus NEXT ulcer*) OR (chronic NEXT wound*) OR (chronic NEXT ulcer*) OR (complex NEXT wound*) OR burn OR burns OR burned OR scald* OR (thermal NEXT injur*)) AND (assess* OR tool* OR measur* OR examin* OR alogrith* OR staging OR classif* OR identif*) | systematic[sb] AND ((skin ulcer* OR foot ulcer* OR diabetic foot OR diabetic feet OR leg ulcer* OR varicose ulcer* OR venous ulcer* OR stasis ulcer* OR ulcer cruris OR ulcus cruris OR crural ulcer* OR arterial ulcer* OR neuropathic ulcer* OR ischaemic wound* OR ischaemic ulcer* OR ischemic wound* OR ischemic ulcer* OR bed sore* OR pressure sore* OR pressure ulcer* OR decubitus ulcer* OR chronic wound* OR chronic ulcer* OR complex wound* OR burn OR burns OR burned OR scald* OR thermal injur*) AND (assess* OR tool* OR measur* OR examin* OR alogrith* OR staging OR classif* OR identif*)) AND (standard* OR regulat* OR audit* OR enforce* OR guideline* OR monitor* OR pathway* OR introduc*) |
| 4a | How does nursing and/or professional skill mix influence wound outcomes in community settings? | (((skin NEXT ulcer*) OR (foot NEXT ulcer*) OR (diabetic NEXT foot) OR (diabetic NEXT feet) OR (leg NEXT ulcer*) OR (varicose NEXT ulcer*) OR (venous NEXT ulcer*) OR (stasis NEXT ulcer*) OR (arterial NEXT ulcer*) OR ((ischaemic or ischemic) NEXT (wound* or ulcer*)) OR (bed NEXT sore*) OR (pressure NEXT sore*) OR (pressure NEXT ulcer*) OR (decubitus NEXT ulcer*) OR (chronic NEXT wound*) OR (chronic NEXT ulcer*) OR (complex NEXT wound*) OR burn OR burns OR burned OR scald* OR (thermal NEXT injur*))AND ((skill OR grade OR staff* OR role) AND (mix OR blend OR level* OR change* OR changing OR model*))) | systematic[sb] AND ((skin ulcer* OR foot ulcer* OR diabetic foot OR diabetic feet OR leg ulcer* OR varicose ulcer* OR venous ulcer* OR stasis ulcer* OR ulcer cruris OR ulcus cruris OR crural ulcer* OR arterial ulcer* OR neuropathic ulcer* OR ischaemic wound* OR ischaemic ulcer* OR ischemic wound* OR ischemic ulcer* OR bed sore* OR pressure sore* OR pressure ulcer* OR decubitus ulcer* OR chronic wound* OR chronic ulcer* OR complex wound* OR burn OR burns OR burned OR scald* OR thermal injur*) AND ((skill OR grade OR staff* OR role) AND (mix OR blend* OR level* OR change* OR changing OR models OR model OR modelling))) |
| 4b | What training is required to best manage patients with complex wounds? | (((skin NEXT ulcer*) OR (foot NEXT ulcer*) OR (diabetic NEXT foot) OR (diabetic NEXT feet) OR (leg NEXT ulcer*) OR (varicose NEXT ulcer*) OR (venous NEXT ulcer*) OR (stasis NEXT ulcer*) OR (arterial NEXT ulcer*) OR ((ischaemic or ischemic) NEXT (wound* or ulcer*)) OR (bed NEXT sore*) OR (pressure NEXT sore*) OR (pressure NEXT ulcer*) OR (decubitus NEXT ulcer*) OR (chronic NEXT wound*) OR (chronic NEXT ulcer*) OR (complex NEXT wound*) OR burn OR burns OR burned OR scald* OR (thermal NEXT injur*))AND ((train* OR educat* OR expertise OR competenc* OR coach* OR instruct* OR guid* OR prepar* OR teach* OR tutor*))) | systematic[sb] AND (wound* OR(skin ulcer* OR foot ulcer* OR diabetic foot OR diabetic feet OR leg ulcer* OR varicose ulcer* OR venous ulcer* OR stasis ulcer* OR ulcer cruris OR ulcus cruris OR crural ulcer* OR arterial ulcer* OR neuropathic ulcer* OR ischaemic wound* OR ischaemic ulcer* OR ischemic wound* OR ischemic ulcer* OR bed sore* OR pressure sore* OR pressure ulcer* OR decubitus ulcer* OR chronic wound* OR chronic ulcer* OR complex wound* OR burn OR burns OR burned OR scald* OR thermal injur*) AND (train* OR educat* OR expertise OR competenc* OR coach* OR instruct* OR guid* OR prepar* OR teach* OR tutor*)) |
| 5 | Do integrated team-based interventions aimed at better communication and collaborative working, improve patient outcomes? | (team OR collaborat*) AND (work OR care OR integrated OR multidisciplinary OR multi-disciplinary OR interdisciplinary OR inter-disciplinary OR interprofessional OR inter-professional) | systematic[sb] AND ((team* OR multidisciplinary OR multi-disciplinary OR interdisciplinary OR interdisciplinary) AND (patient* AND outcome*)) |
| 6 | Does continuing professional development in wound care improve the quality of care and patient outcomes compared with no annual update? | ("continuing professional development" OR CPD OR train* OR educat*) AND ((skin NEXT ulcer*) OR (foot NEXT ulcer*) OR (diabetic NEXT foot) OR (diabetic NEXT feet) OR (leg NEXT ulcer*) OR (varicose NEXT ulcer*) OR (venous NEXT ulcer*) OR (stasis NEXT ulcer*) OR (arterial NEXT ulcer*) OR ((ischaemic or ischemic) NEXT (wound* or ulcer*)) OR (bed NEXT sore*) OR (pressure NEXT sore*) OR (pressure NEXT ulcer*) OR (decubitus NEXT ulcer*) OR (chronic NEXT wound*) OR (chronic NEXT ulcer*) OR (complex NEXT wound*) OR burn OR burns OR burned OR scald* OR (thermal NEXT injur*)) | systematic[sb] AND (CPD OR professional development OR trained OR trains OR training OR educat*) AND ((skin ulcer* OR foot ulcer* OR diabetic foot OR diabetic feet OR leg ulcer* OR varicose ulcer* OR venous ulcer* OR stasis ulcer* OR ulcer cruris OR ulcus cruris OR crural ulcer* OR arterial ulcer* OR neuropathic ulcer* OR ischaemic wound* OR ischaemic ulcer* OR ischemic wound* OR ischemic ulcer* OR bed sore* OR pressure sore* OR pressure ulcer* OR decubitus ulcer* OR chronic wound* OR chronic ulcer* OR complex wound* OR burn OR burns OR burned OR scald* OR thermal injur*)) |
| 7 | What effects do electronic patient records have on patient and service outcomes across a wound care service compared to paper records? | (electronic AND record*) OR e-record* | systematic[sb] AND ((electronic AND record*) OR e-record*) |
| 8 | Which treatments are most effective for over granulation? | over NEXT granulat* OR overgranulat* OR hypergranulat* OR hyper NEXT granulat* | systematic[sb] AND (over or hyper AND granulati*) OR overgranulat* or hypergranulat* |
| 9 | What is the most clinical and cost-effective criteria for referring to specialist services (e.g. tissue viability/podiatry) to ensure appropriate use of resources and referral time? | (specialis* OR podiatr* OR chirop* OR tissue NEXT viability OR TVN OR team* OR clinics OR clinic OR servic*) AND ((skin NEXT ulcer*) OR (foot NEXT ulcer*) OR (diabetic NEXT foot) OR (diabetic NEXT feet) OR (leg NEXT ulcer*) OR (varicose NEXT ulcer*) OR (venous NEXT ulcer*) OR (stasis NEXT ulcer*) OR (arterial NEXT ulcer*) OR ((ischaemic or ischemic) NEXT (wound* or ulcer*)) OR (bed NEXT sore*) OR (pressure NEXT sore*) OR (pressure NEXT ulcer*) OR (decubitus NEXT ulcer*) OR (chronic NEXT wound*) OR (chronic NEXT ulcer*) OR (complex NEXT wound*) OR burn OR burns OR burned OR scald* OR (thermal NEXT injur*)) | systematic[sb] AND (refer* OR Consult*) AND ((specialis* OR podiatr* OR chirop* OR tissue viability OR TVN) AND (skin ulcer* OR foot ulcer* OR diabetic foot OR diabetic feet OR leg ulcer* OR varicose ulcer* OR venous ulcer* OR stasis ulcer* OR ulcer cruris OR ulcus cruris OR crural ulcer* OR arterial ulcer* OR neuropathic ulcer* OR ischaemic wound* OR ischaemic ulcer* OR ischemic wound* OR ischemic ulcer* OR bed sore* OR pressure sore* OR pressure ulcer* OR decubitus ulcer* OR chronic wound* OR chronic ulcer* OR complex wound* OR burn OR burns OR burned OR scald* OR thermal injur*))) |
| 10a and 10 b | How do we differentiate between diabetic foot wounds and pressure ulcers? Does this influence management and outcomes? | ((skin NEXT ulcer*) OR (foot NEXT ulcer*) OR (diabetic NEXT foot) OR (diabetic NEXT feet)) AND ((bed NEXT sore*) OR (pressure NEXT sore*) OR (pressure NEXT ulcer*) OR (decubitus NEXT ulcer*)) | systematic[sb] AND ((skin ulcer* OR foot ulcer* OR diabetic foot OR diabetic feet) AND (bed sore* OR pressure sore* OR pressure ulcer* OR decubitus ulcer*)) |
| 11 | Do patients with venous leg ulceration heal quicker when treated in a dedicated leg ulcer clinic compared with general community clinics? | ((leg NEXT ulcer*) OR (varicose NEXT ulcer*) OR (venous NEXT ulcer*) OR (stasis NEXT ulcer*) OR ((ischaemic or ischemic) NEXT (wound* or ulcer*))) AND (clinic OR clinics) | systematic[sb] AND ((leg ulcer* OR varicose ulcer* OR venous ulcer* OR stasis ulcer* OR ulcer cruris OR ulcus cruris OR crural ulcer* OR ischaemic wound* OR ischaemic ulcer* OR ischemic wound* OR ischemic ulcer*) AND (clinic OR clinics)) |
| 12 | What are the effects of different cleansing agents on infection and healing of wounds in community settings? | ((skin NEXT ulcer*) OR (foot NEXT ulcer*) OR (diabetic NEXT foot) OR (diabetic NEXT feet) OR (leg NEXT ulcer*) OR (varicose NEXT ulcer*) OR (venous NEXT ulcer*) OR (stasis NEXT ulcer*) OR (arterial NEXT ulcer*) OR ((ischaemic or ischemic) NEXT (wound* or ulcer*)) OR (bed NEXT sore*) OR (pressure NEXT sore*) OR (pressure NEXT ulcer*) OR (decubitus NEXT ulcer*) OR (chronic NEXT wound*) OR (chronic NEXT ulcer*) OR (complex NEXT wound*) OR burn OR burns OR burned OR scald* OR (thermal NEXT injur*)) AND clean* | systematic[sb] AND ((skin ulcer* OR foot ulcer* OR diabetic foot OR diabetic feet OR leg ulcer* OR varicose ulcer* OR venous ulcer* OR stasis ulcer* OR ulcer cruris OR ulcus cruris OR crural ulcer* OR arterial ulcer* OR neuropathic ulcer* OR ischaemic wound* OR ischaemic ulcer* OR ischemic wound* OR ischemic ulcer* OR bed sore* OR pressure sore* OR pressure ulcer* OR decubitus ulcer* OR chronic wound* OR chronic ulcer* OR complex wound* OR burn OR burns OR burned OR scald* OR thermal injur* OR wound*) AND clean*) |
| 13 | What are the clinical and cost effective methods for managing an excess of wound exudate? | Exud* | systematic[sb] AND ((exudat*) AND (skin ulcer* OR foot ulcer* OR diabetic foot OR diabetic feet OR leg ulcer* OR varicose ulcer* OR venous ulcer* OR stasis ulcer* OR ulcer cruris OR ulcus cruris OR crural ulcer* OR arterial ulcer* OR neuropathic ulcer* OR ischaemic wound* OR ischaemic ulcer* OR ischemic wound* OR ischemic ulcer* OR bed sore* OR pressure sore* OR pressure ulcer* OR decubitus ulcer* OR chronic wound* OR chronic ulcer* OR complex wound* OR burn OR burns OR burned OR scald* OR thermal injur*)) |
| 14 | Does sharp debridement speed up wound healing in chronic wounds compared with dressings (HCL, hydrogels etc)? | Debrid* | systematic[sb] AND ((sharp* OR surg* OR mechanical) AND debrid*) |
| 15a and 15b | How should we identify where biofilm is impeding wound healing? What is the best way to manage a biofilm? | biofilm* OR slime | systematic[sb] AND ((biofilm* OR Slime) AND (skin ulcer* OR foot ulcer* OR diabetic foot OR diabetic feet OR leg ulcer* OR varicose ulcer* OR venous ulcer* OR stasis ulcer* OR ulcer cruris OR ulcus cruris OR crural ulcer* OR arterial ulcer* OR neuropathic ulcer* OR ischaemic wound* OR ischaemic ulcer* OR ischemic wound* OR ischemic ulcer* OR bed sore* OR pressure sore* OR pressure ulcer* OR decubitus ulcer* OR chronic wound* OR chronic ulcer* OR complex wound* OR burn OR burns OR burned OR scald* OR thermal injur* OR wound*)) |
| 16 | How do we promote adherence to interventions and health behaviours in people at high risk of foot problems? | ((promot* OR prevent* OR adher* OR complian* OR concord* OR sustain* OR maintain*) AND (intervent* OR treat* OR behav*)) AND ((skin NEXT ulcer*) OR (foot NEXT ulcer*) OR (diabetic NEXT foot) OR (diabetic NEXT feet) OR (neuropathic NEXT ulcer*) OR (risk AND (foot OR feet))) | systematic[sb] AND ((promot* OR prevent* OR adher* OR complian* OR concord* OR sustain* OR maintain*) AND (intervent* OR treat* OR behav*)) AND ((foot ulcer* OR diabetic foot OR diabetic feet OR neuropathic ulcer*) OR (risk AND (foot OR feet))))) |
| 17 | Do anti-microbial containing wound dressings heal infected wounds more quickly than oral antimicrobials? | ((antimicro* OR antibact* OR antifung* OR antivir* OR antibioti* OR antisepti*) AND dress*) AND (infec* AND (wound* OR ulcer* OR burn* OR scald* OR sore*)) | systematic[sb] AND ((((Topical OR dress*) AND oral) AND antimicrob* OR antibiotic OR antibiotics OR antifung* OR antisept*) AND ((infect* AND (skin ulcer* OR foot ulcer* OR diabetic foot OR diabetic feet OR leg ulcer* OR varicose ulcer* OR venous ulcer* OR stasis ulcer* OR ulcer cruris OR ulcus cruris OR crural ulcer* OR arterial ulcer* OR neuropathic ulcer* OR ischaemic wound* OR ischaemic ulcer* OR ischemic wound* OR ischemic ulcer* OR bed sore* OR pressure sore* OR pressure ulcer* OR decubitus ulcer* OR chronic wound* OR chronic ulcer* OR complex wound* OR burn OR burns OR burned OR scald* OR thermal injur* OR wound*))) |
| 18 | Does a prescribed two week treatment plan, using the same type of dressing, affect healing outcomes versus ad-hoc dressing selection? | (plan* OR prescribe* OR prescribed OR time OR timing OR duration OR leav* OR left OR remain* OR Consistent* OR same OR repeat*OR unchang* OR unalter* OR routine* OR persist* OR maintain*OR retain* OR frequen*) AND (wound* OR ulcer* OR dress*) | Systematic[sb] AND ((skin ulcer* OR foot ulcer* OR diabetic foot OR diabetic feet OR leg ulcer* OR varicose ulcer* OR venous ulcer* OR stasis ulcer* OR ulcer cruris OR ulcus cruris OR crural ulcer* OR arterial ulcer* OR neuropathic ulcer* OR ischaemic wound* OR ischaemic ulcer* OR ischemic wound* OR ischemic ulcer* OR bed sore* OR pressure sore* OR pressure ulcer* OR decubitus ulcer* OR chronic wound* OR chronic ulcer* OR complex wound* OR burn OR burns OR burned OR scald* OR thermal injur* OR wound*) AND ((dress* OR treat* OR intervent*) AND (plan OR plans OR planned OR planning OR prescribe OR prescribes OR prescription OR prescriptions OR prescribing OR prescribed OR time OR timing OR duration OR leave OR leaves OR leaving OR left OR remain or remaining OR remained OR Consistent OR consistently OR consistency OR same OR repeats OR repeating OR repeated OR unchanged OR unchanging OR unaltered OR routine OR routinely OR persistent OR persisting OR persisted OR persist OR maintained OR maintaining OR maintain OR retained OR retain OR retaining OR frequency OR frequent))) |
| 19 | What is the best way of cleaning venous leg ulcers in terms of promoting healing and preventing infection? | clean* AND ((leg NEXT ulcer*) OR (varicose NEXT ulcer*) OR (venous NEXT ulcer*) OR (stasis NEXT ulcer*) OR ((ischaemic or ischemic) NEXT (wound* or ulcer*))) | systematic[sb] AND (clean* AND (leg ulcer* OR varicose ulcer* OR venous ulcer* OR stasis ulcer* OR ulcer cruris OR ulcus cruris OR crural ulcer* OR ischaemic wound* OR ischaemic ulcer* OR ischemic wound* )) |
| 20 | Do psychological interventions (i.e., aimed at changing health beliefs and behaviours) improve the healing/reduce the incidence of ulcers on the feet of people with diabetes? | diabet* AND (foot OR feet OR ulcer*) | systematic[sb] AND ((brief intervention OR motivational interview* OR psychological therap* OR psychological intervent* OR counsel* OR talking therap* OR iapt) AND ((diabetic OR diabete* AND (foot OR feet OR ulcer*) OR neuropath* ulcer*))) |
| 21 | How can accurate detection of clinical infection be facilitated across different skill mixes? | Infect* AND (detect* OR assess* OR identif* OR disting* OR diagnos* OR determin* OR screen*) | systematic[sb] AND ((infect* AND (detect* OR assess* OR identif* OR disting* OR diagnosis OR diagnosed OR determin* OR screen*)) AND ((skill OR grade OR staff* OR role OR team) AND (mix OR blend OR level OR changes OR change OR changing OR model OR reprofil*) OR task shift*)) |
| 22 | What should be used for infected wounds when the bacteria are resistant to antibiotics? | (Wound* OR burn* OR ulcer* OR sore* OR scald*) AND (resistan* AND antibiotic*) | Systematic[sb] AND ((Wound* OR Burn* OR UIcer* OR sore*) AND (resistan* AND antibiotic*)) |
| 23 | Does off-loading for people with foot wounds Improve wound healing compared with usual (or increased) activity? | (Off NEXT load* OR off-load* OR rest* OR pressur* or elevat*) AND ((foot OR feet) AND wound* OR ulcer* OR sore*) | systematic[sb] AND ((Off load* OR off-load* OR rest* OR elevat*) AND ((wound* OR burn* OR ulcer* OR sore*) AND (foot OR feet))) |
| 24 | What impact do walk in centres have on patients outcomes versus treatment room clinics? | “Walk in” OR Walk-in OR WIC OR “drop in” OR drop-in OR (community NEXT clinic*) OR (treatment NEXT room) | systematic[sb] AND ((WiC OR "walk in" OR walk-in OR “drop in” OR drop-in) AND (center OR centre OR clinic OR centers OR centres OR clinics)) |
| 25 | Does stopping packing a sinus wound when it has healed to 1cm depth and then treating with medical honey speed wound healing compared with usual care? | (pack* OR honey) AND (sinus OR wound*) | systematic[sb] AND ((Pack* OR honey) AND (sinus OR wound*)) |
